# GPR18-Mediated Relaxation of Human Isolated Pulmonary Arteries

**DOI:** 10.3390/ijms23031427

**Published:** 2022-01-26

**Authors:** Hanna Kozłowska, Barbara Malinowska, Marta Baranowska-Kuczko, Magdalena Kusaczuk, Miłosz Nesterowicz, Mirosław Kozłowski, Christa E. Müller, Katarzyna Kieć-Kononowicz, Eberhard Schlicker

**Affiliations:** 1 Department of Experimental Physiology and Pathophysiology, Medical University of Białystok, ul. Mickiewicza 2A, 15-222 Białystok, Poland; bmalin@umb.edu.pl (B.M.); mabar@umb.edu.pl (M.B.-K.); 2 Department of Clinical Pharmacy, Medical University of Białystok, ul. Mickiewicza 2A, 15-222 Białystok, Poland; 3 Department of Pharmaceutical Biochemistry, Medical University of Białystok, ul. Mickiewicza 2A, 15-222 Białystok, Poland; mkusaczuk@wp.pl; 4 Department of Thoracic Surgery, Medical University of Białystok, ul. M.C. Skłodowska 4A, 15-276 Białystok, Poland; miloszn1999@gmail.com (M.N.); miroslaw.kozlowski@umb.edu.pl (M.K.); 5 Department of Pharmaceutical & Medicinal Chemistry, Pharmaceutical Institute, PharmaCenter Bonn, University of Bonn, An der Immenburg 4, 53121 Bonn, Germany; christa.mueller@uni-bonn.de; 6 Research Training Group 1873, University of Bonn, Venusberg-Campus 1, 53127 Bonn, Germany; 7 Department of Technology and Biotechnology of Drugs, Faculty of Pharmacy, Jagiellonian University, Medical College, ul. Medyczna 9, 30-688 Kraków, Poland; mfkonono@cyf-kr.edu.pl; 8 Department of Pharmacology and Toxicology, University of Bonn, Venusberg-Campus 1, 53127 Bonn, Germany; e.schlicker@uni-bonn.de

**Keywords:** GPR18 ligands, human pulmonary artery, vasorelaxation activity

## Abstract

GPR18 receptor protein was detected in the heart and vasculature and appears to play a functional role in the cardiovascular system. We investigated the effects of the new GPR18 agonists PSB-MZ-1415 and PSB-MZ-1440 and the new GPR18 antagonist PSB-CB-27 on isolated human pulmonary arteries (hPAs) and compared their effects with the previously proposed, but unconfirmed, GPR18 ligands NAGly, Abn-CBD (agonists) and O-1918 (antagonist). GPR18 expression in hPAs was shown at the mRNA level. PSB-MZ-1415, PSB-MZ-1440, NAGly and Abn-CBD fully relaxed endothelium-intact hPAs precontracted with the thromboxane A_2_ analog U46619. PSB-CB-27 shifted the concentration-response curves (CRCs) of PSB-MZ-1415, PSB-MZ-1440, NAGly and Abn-CBD to the right; O-1918 caused rightward shifts of the CRCs of PSB-MZ-1415 and NAGly. Endothelium removal diminished the potency and the maximum effect of PSB-MZ-1415. The potency of PSB-MZ-1415 or NAGly was reduced in male patients, smokers and patients with hypercholesterolemia. In conclusion, the novel GPR18 agonists, PSB-MZ-1415 and PSB-MZ-1440, relax hPAs and the effect is inhibited by the new GPR18 antagonist PSB-CB-27. GPR18, which appears to exhibit lower activity in hPAs from male, smoking or hypercholesterolemic patients, may become a new target for the treatment of pulmonary arterial hypertension.

## 1. Introduction

GPR18 is an orphan G protein-coupled receptor, which, although showing little structural similarity to cannabinoid CB_1_ and CB_2_ receptors (identity of amino acid sequence of 13 and 8%, respectively; [1]), responds to the natural cannabinoid Δ^9^-tetrahydrocannabinol (THC). In the mouse, GPR18 is located in the central nervous system, the gastrointestinal tract, in the lymphoid system [2,3], in peripheral blood leukocytes, several hematopoietic cell lines [4,5] and in the eye [6,7]. GPR18 expression has recently been verified in skeletal muscle from obese rats [8]. In humans, this receptor is expressed in the brain including the hypothalamus [9], in cells of the immune system [4,5], and in the colon [10]. GPR18 is also expressed in cultured cells, including human lymphoid cells [4], HEC-1B endometrial cells [11], BV2 murine microglial cells [12], and metastatic melanoma [13]. Although the (patho)physiological role of GPR18 is controversial and unclear, it is believed that it may be involved in immunological processes, inhibition of inflammatory reactions including intestinal inflammation [10,14] and in lowering intraocular pressure in mice [7,15].

GPR18 receptor protein was also detected in the cardiovascular system, including the heart [16], isolated vessels and endothelial cells of mesenteric arteries [17], retinal vessels of the rat [6], pulmonary arteries [18], and placental vascular smooth muscle of humans [19] as well as various vascular endothelial cell lines [6,20,21]. It was also found to be expressed in the rat’s rostral ventrolateral medulla (RVLM), a brain region involved in the regulation of cardiovascular function [9]. 

Some functional studies indeed suggest that GPR18 plays a role in the cardiovascular system. Thus, chronic administration of an agonist to the RVLM was reported to lower mean blood pressure, suppress the cardiac sympathetic dominance and improve left ventricular function in conscious rats [16], and to display similar cardioprotective effects in a rat model of streptozotocin-induced diabetes [22]. Moreover, mesenteric vasodilation and hypotension in mice lacking CB_1_/CB_2_ receptors has been shown [23], and an endothelium-dependent vasorelaxation has been described in small mesenteric arteries of the rat [17,24] and in human pulmonary arteries (hPAs; [25,26]). All of these data are based on cannabinoids such as THC and abnormal cannabidiol (Abn-CBD), which were proposed to act as agonists, and O-1918, proposed to act as an antagonist, both in HEK293-GPR18 transfected cells [11] and in various vascular preparations. The receptor involved in the vasodilation of mesenteric arteries of rodents was described earlier than GPR18 and labeled “endothelial cannabinoid receptor” [27]. Some authors suggested that the latter receptor, which is not defined in terms of molecular biology, may be GPR18 [12]. 

There are, however, difficulties in the unambiguous assessment of GPR18 since the agonistic or antagonistic effects of the cannabinoids mentioned above (except for THC) were not confirmed [28,29,30]. This also holds true for *N*-arachidonyl-glycine (NAGly; [31]), which was proposed as the endogenous ligand of GPR18 [4]). Interesting enough, NAGly activates (1) large calcium-dependent potassium channels (BK_Ca_; [17,32], (2) small calcium-dependent potassium channels (SK_Ca;_ [6], and (3) the Na^+^/Ca^2+^ exchanger (NCX) leading to NO production [24]. Each of the latter three mechanisms would lead to vascular relaxation.

Recently, novel ligands for GPR18 have become available, including the agonists PSB-MZ-1415 and PSB-MZ-1440 and the antagonist PSB-CB-27 [29,33]. Taking into account the fact that GPR18 protein is expressed in the human pulmonary artery [18], the aim of the present study was to investigate the effects of the three novel GPR18 ligands on isolated hPAs and to compare their effects with the previously proposed, but unconfirmed, GPR18 ligands NAGly, Abn-CBD, and O-1918. In addition, we searched for *gpr18* mRNA, using reverse transcriptase polymerase chain reaction (RT-PCR).

## 2. Results

### 2.1. General

The vasorelaxant effects of agonists on hPAs were studied in vascular rings precontracted with U46619 (0.01 μM, a concentration approximately equivalent to its EC_60_). In all experimental groups, the U46619-induced increase in tone was comparable (Table 1) and the U46619-induced contractions were not affected by 30 min incubation periods with the antagonists or their vehicles.

### 2.2. Influence of Agonists on U46619-Precontracted Human Pulmonary Arteries

**Figure 1:**
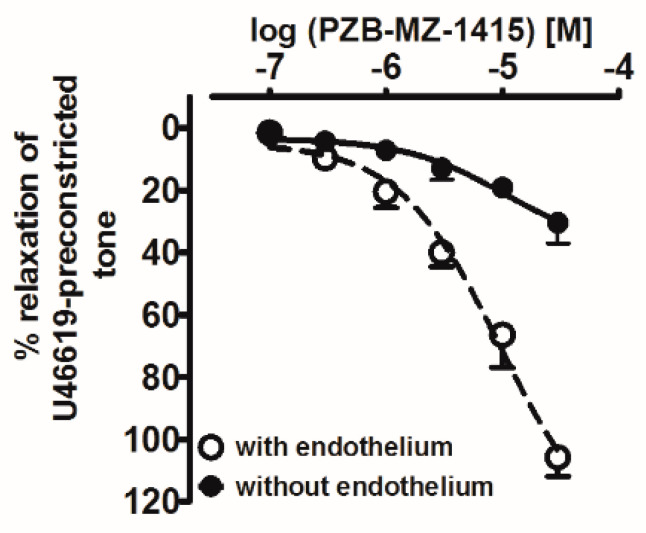
Influence of endothelium removal on the vasorelaxation induced by PSB-MZ-1415 in isolated human pulmonary arteries. Results are expressed as the percentage relaxation of the isometric contraction induced by U46619 (*n* = 3 per group) in comparison to the respective control.

**Figure 2:**
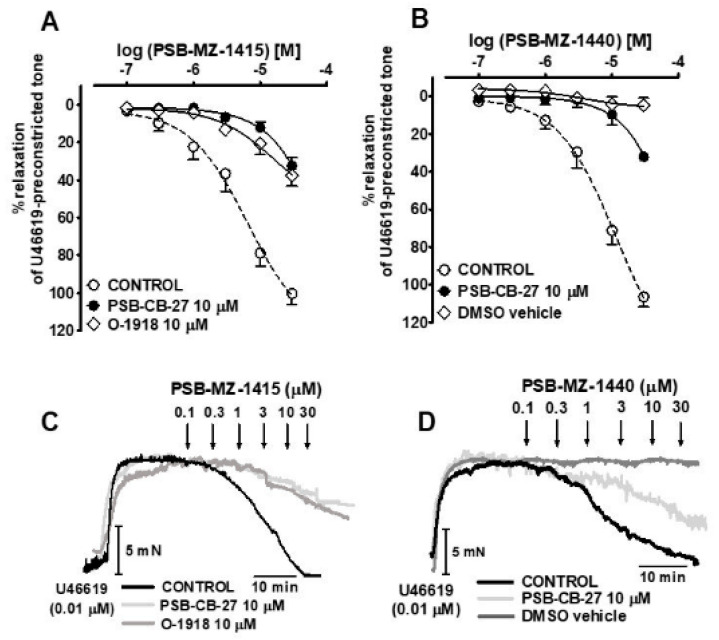
Influence of PSBCB–27 (**A**–**D**) and O–1918 (**A**,**C**) on the vasorelaxation induced by PSB–MZ–1415 (**A**,**C**) and PSB–MZ–1440 (**B**,**D**) in endothelium-intact isolated human pulmonary arteries. Concentration-response curves (mean ± SEM of 5–7 tissue samples) and representative original traces of the PSB–MZ–1415–(**A**,**C**) or PSB–MZ–1440–(**B**,**D**) induced relaxation (also in the presence of PSB–CB–27 or O–1918) are shown in the upper and lower panels, respectively. In (**A**,**B**), the results are expressed as the percentage relaxation of the isometric contraction induced by U46619. See Table 1 and Table 2 for the statistical analysis and for potencies. CONTROL refers to the effects of the agonists in the absence of the particular antagonist. The effects of increasing concentrations of DMSO (max. < 1%), given repeatedly, are shown as well (**B**,**D**). Arrows show the moment of application of a particular concentration of PSB–MZ–1415 or PSB–MZ–1440.

**Figure 3:**
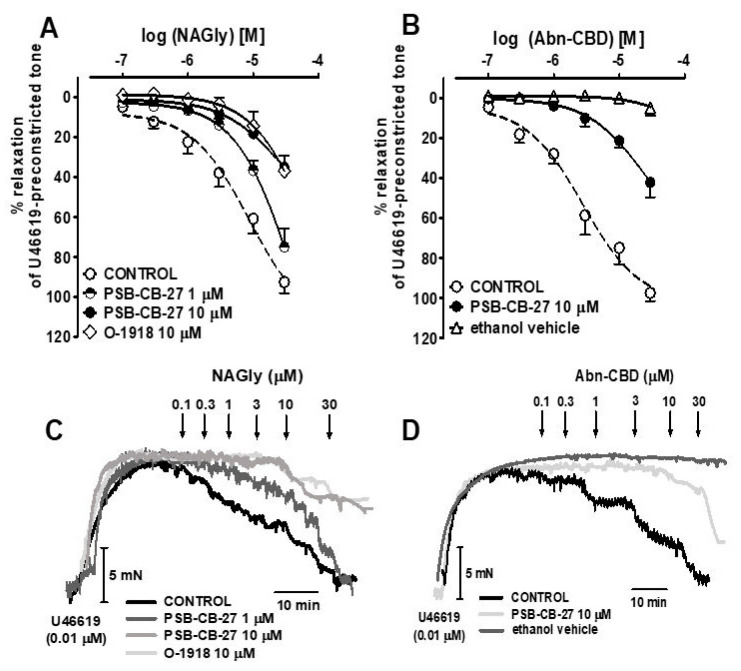
Influence of PSB–CB–27 (**A**–**D**) and O–1918 (**A**,**C**) on the vasorelaxation induced by NAGly (**A**,**C**) and Abn–CBD (**B**,**D**) in endothelium-intact isolated human pulmonary arteries. Concentration-response curves (mean ± SEM of 7–13 tissue samples) and representative original traces of the NAGly–(**A**,**C**) or Abn–CBD–(**B**,**D**) induced relaxation (also in the presence of PSB–CB–27 or O–1918) are shown in the upper and lower panels, respectively. In (**A**,**B**), the results are expressed as the percentage relaxation of the isometric contraction induced by U46619. See Table 1 and Table 2 for the statistical analysis and for potencies. CONTROL refers to the effects of the agonists in the absence of the particular antagonist. The effects of increasing concentrations of ethanol (max. < 0.6%), given repeatedly, are shown as well (**B**,**D**). Arrows show the moment of application of a particular concentration of NAGly or Abn–CBD.

The previously proposed GPR18 agonists NAGly and Abn-CBD, and the novel GPR18 agonists PSB-MZ-1415 and PSB-MZ-1440, were studied in a concentration range of 0.01–30 μM. PSB-MZ-1415 produced a full concentration-dependent relaxation of endothelium-intact isolated hPAs precontracted with U46619; endothelial removal reduced both its potency and its maximum effect (Figure 1 and Figure 2A,C, Table 1). PSB-MZ-1440, NAGly and Abn-CBD also fully relaxed endothelial-dependent and U46619-precontracted hPAs (Figure 2B and Figure 3A,B; for original traces see Figure 2D and Figure 3C,D). The rank order of potencies (according to their pEC_50_ values) was Abn-CBD ≥ PSB-MZ-1415 ≥ NAGly ≥ PSB-MZ-1440 (for pEC_50_, pEC_25_, and R_max_, see Table 1). Repeated administration of the vehicles DMSO and ethanol had a very slight vasorelaxant effect only (Figure 2 and Figure 3, Table 1).

### 2.3. Interaction of PSB-CB-27 and O-1918 with the Agonist-Induced Relaxation in Human Pulmonary Arteries

To investigate the involvement of GPR18 in the effect of the four agonists, their interaction with the new GPR18 antagonist PSB-CB-27 was studied and compared to the previously proposed antagonist O-1918. The concentration-response curves for the PSB compounds are shown in Figure 2, and those of NAGly and Abn-CBD are depicted in Figure 3; original traces are presented in the bottom part of the two figures. pEC_25_ values in the absence and presence of the antagonists and their apparent pA_2_ values are given in Table 1 and Table 2, respectively.

PSB-CB-27 was studied against each of the four GPR18 agonists at 10 µM and, in the case of NAGly, also at 1 µM. The GPR18 antagonist PSB-CB-27 (1 and 10 µM) shifted the concentration-response curve (CRC) for NAGly to the right by a factor of 4 and 9.5, respectively. In the case of Abn-CBD, PSB-MZ-1415, and PSB-MZ-1440, PSB-CB-27 (10 µM) elicited rightward shifts of 25-, 16- and 25-fold, respectively. The antagonist O-1918 (10 μM) was studied against NAGly and PSB-MZ-1415 only and caused a 12.5- and 9.5-fold rightward shift of their CRCs, respectively. 

### 2.4. Post hoc Analysis of the Influence of Co-Morbidities on PSB-MZ-1415- and NAGly-Mediated Vasorelaxation in Human Pulmonary Arteries

**Figure 4:**
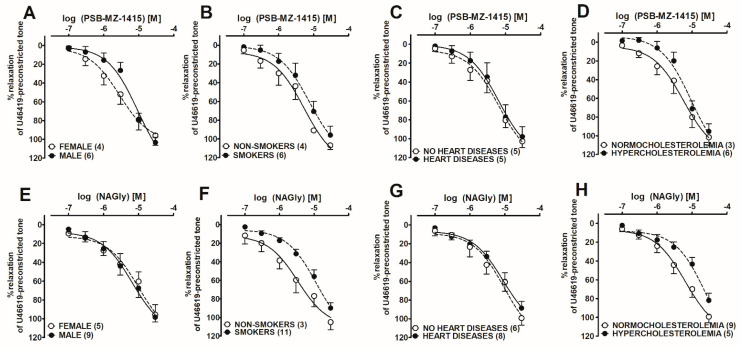
Post hoc analysis of the effect of sex and co-morbidities on PSB–MZ–1415–(**A**–**D**) and NAGly–(**E**–**H**) mediated vasorelaxation in endothelium-intact isolated human pulmonary arteries. The results are presented as the mean ± SEM of n (given in parentheses) tissue samples for each curve. Student’s *t*-test for unpaired data was used to compare the curves (pEC50 values). (**A**) *p* = 0.0096; (**B**) *p* = 0.2133; (**C**) *p* = 1.0; (**D**) *p* = 0.2524; (**E**) *p* = 0.2197; (**F**) *p*= 0.0174; (**G**) *p* = 1.0; (**H**) *p* = 0.0022. See Table 3 for further details.

Post hoc analysis was performed to establish selected relationships between PSB-MZ-1415 and NAGly responses and patient characteristics (Figure 4, Table 3). The potency of NAGly (pEC_50_) was reduced in smokers (4.9 ± 0.1) compared to non-smokers (5.5 ± 0.2, *p* < 0.05; Figure 4F, Table 3), and in patients with hypercholesterolemia (4.6 ± 0.1) compared to normocholesterolemic patients (5.2 ± 0.1, *p* < 0.01; Figure 4H, Table 3), whereas the potency of PSB-MZ-1415 was reduced in males (5.0 ± 0.1) compared to females (5.5 ± 0.1, *p* < 0.01; Figure 4A) and showed a tendency towards a decrease in smokers and in patients with hypercholesterolemia (Figure 4B,D; Table 3). Heart diseases had no influence on the potency of these drugs (Figure 4C,G). We could not perform an analysis for all diseases and for PSB-MZ-1440 and Abn-CBD, as patient numbers were too small for adequate statistical analysis.

### 2.5. gpr18 mRNA Expression in Human Bronchioles and Pulmonary Arteries 

**Figure 5:**
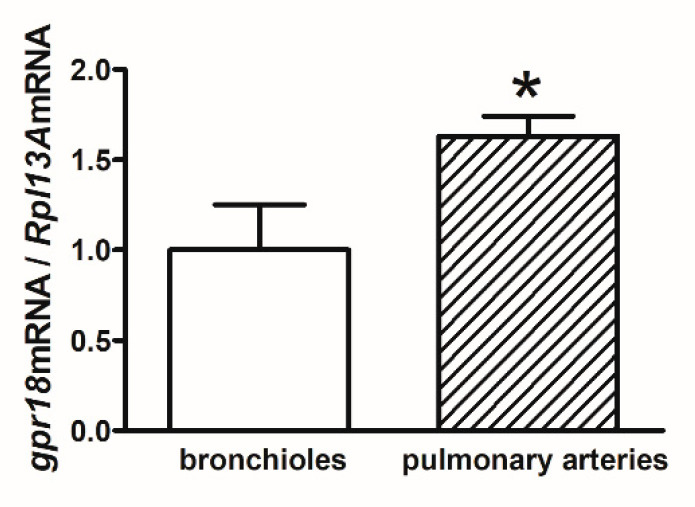
Relative mRNA expression level of *gpr18* evaluated by real-time qPCR assay in isolated human bronchioles and pulmonary arteries. The RNA isolated from bronchioles was used as the control, and its expression was set 1.0. Results are shown as a relative fold change in mRNA expression in comparison to control. The results are presented as the mean ± SEM of 6 tissue samples. * *p* < 0.05.

In order to confirm *gpr18* mRNA distribution in lung tissue and to investigate whether *gpr18* presents a differential expression pattern in bronchioles and pulmonary arteries, a real-time quantitative PCR (qPCR) analysis of the gene transcript was performed. The results showed that *gpr18* mRNA expression was higher in pulmonary arteries compared to bronchioles (Figure 5). 

## 3. Discussion

Our previous studies on the human pulmonary artery revealed that several cannabinoids including Abn-CBD [25], anandamide (AEA; [26]), cannabidiol [18], 2-arachidonoylglycerol [34], and virodhamine [35] elicit an endothelium-dependent vasorelaxation. The effect of Abn-CBD, AEA, and virodhamine is antagonized by O-1918 [25,26,35], suggesting the involvement of the so-called endothelial cannabinoid receptor. There is some evidence that the latter receptor (which is not defined in terms of molecular biology) is identical to the orphan receptor GPR18 [12]. Using an immunohistochemical technique, we detected GPR18 protein in the endothelial and muscular layer of the human pulmonary artery [18]. In the present study, we examined whether also GPR18 mRNA is detectable in the human pulmonary artery and compared the effects of NAGly, a previously proposed, but unconfirmed, endogenous agonist of GPR18 [4], of two newly developed GPR18 agonists, and of a newly developed GPR18 antagonist [33] to the effects of Abn-CBD and O-1918. 

Using the RT-PCR technique, we were indeed able to detect *gpr18* mRNA in hPAs; *gpr18* mRNA expression in pulmonary arteries is more abundant than in bronchioles. These differences in regional mRNA expression may not link to the same changes in protein. Following the detection of *gpr18* mRNA and protein [18], further experiments dedicated to the third level of GPR18, i.e.**,** to its function, were carried out on isolated hPAs, which were contracted by the thromboxane A_2_ analogue U46619. Thromboxane A_2_ receptors are present in large amounts in pulmonary vessels [34], and U46619 has been used to study cannabinoid ligands in our previous work [18,34]. 

The new agonists PSB-MZ-1415 and PSB-MZ-1440 were optimized based on an initial lead [36]. Their potency (pEC_50_) at GPR18 was studied in β-arrestin recruitment assays in CHO cells stably expressing the hGPR18 and amounted to 7.7 and 7.2, respectively (publication in preparation). However, the coupling of GPR18 is currently unknown. Early reports on G_i_ protein coupling [4] could not be unambiguously confirmed so far [36]. The reason why the pEC_50_ values of the newly developed GPR18 agonists in a cellular functional assay (7.2–7.7) were relatively high when compared to relaxation of hPAs (4.9–5.2) is unclear but not surprising, given the more complex system.

For the sake of comparison, we also studied NAGly and Abn-CBD, the agonistic effect of which was shown in HEK293-GPR18 transfected cells [12], but later disputed by other groups [28,29,30,31]. The novel and older compounds resembled each other in terms of maximum effect and potency. All investigated agonists induced a concentration-dependent full relaxation. The concentration range (0.1–30 μM) was the same for each of the four compounds. The potencies (pEC_50_) of the four drugs in the hPAs were Abn-CBD (5.5) ≥ PSB-MZ-1415 (5.2) ≥ NAGly (5.1) ≥ PSB-MZ-1440 (4.9); differences were not statistically significant. NAGly and Abn-CBD had been previously studied also in precontracted rat small mesenteric arteries [17,27], which they almost fully relaxed, exhibiting pEC_50_ values of ~ 5.7. 

In order to further clarify whether GPR18 is involved in the relaxation of hPAs, studies were carried out with the new GPR18 antagonist PSB-CB-27 [33]. This compound (inactive at hCB_1_ and hCB_2_ receptors, pIC_50_ < 5), exhibits a pIC_50_ of 6.2 at the hGPR18 in the β-arrestin recruitment assay [33]. Its antagonistic potency is identical to the apparent pA_2_ value (6.2–6.3) against the two novel GPR18 agonists obtained in hPAs. This suggests that the receptor under consideration is a GPR18 since, in the case of antagonists, only the interaction between agonist and antagonist at the receptor level is measured and values obtained in different experimental models should be very similar. Interesting enough, the apparent pA_2_ of PSB-CB-27 against the two other agonists Abn-CBD and NAGly was also 6.2–6.3. These results are compatible with the view, but do not prove, that in our experimental model also the latter two agonists activate GPR18. 

The antagonist at the endothelial cannabinoid receptor, O-1918, had a pA_2_ of 6.0 (mean of the values against PSB-MZ-1415 and NAGly), which is in the middle of the range of pA_2_ values obtained for the vasorelaxant effect in various vascular preparations including hPAs ([25]—5.1, [35]—6.3, [26]—5.6), rat pulmonary arteries ([37]—5.7) and mesenteric arteries ([17]—6.7, [27]—5.8). Moreover, we found that vasorelaxation to PSB-MZ-1415 was strongly reduced in endothelium-denuded hPAs, indicating that PSB-MZ-1415 can act endothelium-dependently and, to a lesser extent, endothelium-independently. Our results are similar to those obtained by Kotańska et al. 2021 [38], who found that another GPR18 receptor agonist, PSB-KD-107, caused endothelium-dependent vasorelaxation of the rat aorta.

While GPR18 mRNA, protein expression, and function have been shown in hPAs, the question arises whether this receptor has practical relevance. Post hoc analysis revealed that the potency of the agonists for GPR18-related relaxation was reduced in males, by smoking, and hypercholesterolemia compared to respective controls. This is in line with the fact that smoking and hypercholesterolemia are well-known important risk factors for endothelial dysfunction [39]. Pulmonary arterial hypertension can be elicited by a multitude of pathophysiological alterations [40], and GPR18 agonists may become an additional strategy for its treatment, thereby adding to the armamentarium of drugs, including prostacyclin analogues, endothelin receptor antagonists, phosphodiesterase 5 inhibitors, and soluble guanylate cyclase stimulators [41]. Interestingly, anti-inflammatory and anti-nociceptive activities of PSB-MZ-1415 have been shown in animal models of intestinal inflammation and inflammatory pain [10], and beneficial effects of a GPR18 agonist (PSB-MZ-1415) and of GPR18 antagonists (PSB-CB-5 and PSB-CB-27) on food intake and body weight gain in rats have also been reported [42]. 

## 4. Materials and Methods

### 4.1. Tissue Preparation

Experimental protocols were approved by the Human Ethics Committee of the Medical University of Białystok, Poland (R-I-002/59/2017). The tissue donors provided written informed consent for the use of their blood vessels. 

Lung tissue was received from 28 patients (19 men and 9 women; patient characteristics are shown in Table 3) undergoing lobectomy or pneumonectomy during the resection of lung carcinoma or tuberculoma with a surgical margin of healthy tissue. Before the operation, they received cephalosporins and heparin as anti-infection and antithrombotic prophylaxis, respectively. Anesthesia was induced intravenously by etomidate and maintained with inhaled sevoflurane. Pancuronium and sufentanil were applied for muscle relaxation and analgesia, respectively. Isolation of the human pulmonary arteries has been described previously [18]. Arterial rings (3–5 mm in length and 2–4 mm in outer diameter) were mounted in 10 mL organ baths containing Tyrode’s solution (for composition, see [18]) gassed with 95% O_2_ and 5% CO_2_ (37 °C, pH 7.4). The hPAs were allowed to equilibrate for 90 min at a resting tension of ~20–25 mN. After the equilibration period, all rings were exposed to high KCl (60 mM) to establish tissue viability. Then, rings were exposed to phenylephrine (1 µM) followed by the induction of at least 80% relaxation in response to acetylcholine (1 µM) to verify if the endothelium was intact. For some experiments, the endothelium was removed by gently rubbing off the intima. Successful endothelial denudation was confirmed by the absence of acetylcholine-induced vasorelaxation.

Muscle tension was recorded by a force displacement transducer (BIO-SYS-TECH, Białystok, Poland) [18].

### 4.2. Vasorelaxation Study

In each individual preparation, only one CRC was determined. All experiments were performed in paired vessels, i.e., the effect of a drug was studied in one vessel and another vessel from the same patient served as a control. 

Human pulmonary arteries were sub-maximally contracted with U46619 (a prostanoid TP receptor agonist) at 0.01 µM, which is approximately equivalent to its EC_60_. After a stable level of tone was maintained, CRCs were generated by the cumulative application of NAGly, Abn-CBD, PSB-MZ-1415, PSB-MZ-1440, or their vehicle. When necessary, tissues were pre-treated for 30 min with one of the following antagonists: PSB-CB-27 (1 or 10 μM; a novel GPR18 receptor antagonist) or O-1918 (10 μM; a previously proposed antagonist). 

In vehicle-treated tissues, identical volumes of the respective vehicles of the agonists or antagonists were administered (dimethyl sulfoxide (DMSO) or ethanol, see below).

### 4.3. RNA Isolation and Gene Expression Analysis

Specimens of lung and bronchial tissues from 6 patients (from the above-mentioned group of 25 patients) were immediately flash-frozen in liquid nitrogen and stored at −80 °C. Approximately 5–10 mg of each tissue were taken to perform RNA purification. Samples were finely ground to a powder with a chilled stainless-steel mortar and pestle. Total RNA was isolated according to the procedure described previously [43]. Briefly, the ReliaPrep RNA Tissue Miniprep System (Promega, Madison, WI, USA) was used for the purification procedure, and traces of genomic DNA were excluded by DNase I treatment following the manufacturer’s instructions. Spectrophotometric measurements (A260/A280) were carried out to assess the quantity and quality of the extracted RNA (NanoPhotometer, Implen, München, Germany). Synthesis of the cDNA was performed using the High Capacity RNA-to-cDNA Kit (Applied Biosystems, Foster City, CA, USA) according to the manufacturer’s protocol. Briefly, 0.5 µg of purified total RNA were used in a 20 µL reaction mixture containing random octamers, oligo dT-16 primers, dNTPs, and MuLV reverse transcriptase (RT). Two microliters of cDNA served as a template for real-time qPCR reactions. Amplification of the product was performed using SsoAdvanced Universal SYBR Green Supermix (Bio-Rad, Hercules, CA, USA). A pre-designed set of primers for human *Grp18* gene was purchased from Bio-Rad (GPR18 PrimePCR™ PreAmp for SYBR^®^ Green Assay (accession no. NC_000013.10)). As an internal control, two reference genes, *Rpl13A* and *Hprt1*, were tested, and *Rpl13A* was chosen for further analysis. The sequences of the housekeeping genes were as previously described: *Rpl13A* sense 5′-CTATGACCAATAGGAAGAGCAACC-3′, antisense 5′-GCAGAGTATATGACCAGGT-GGAA-3′ [44], and *Hprt1* sense 5′-TGCTCGAGATGTGATGAAGC-3′, antisense 5′-AATCCAGCAGGTCAGCAAAG-3′ [45]. Primer accuracy was checked by using the Primer-BLAST tool (http://www.ncbi.nlm.nih.gov/tools/primer-blast, accessed on 24 January 2022). The following reaction parameters were applied: initial denaturation at 98 °C for 30 s, followed by 40 cycles at 95 °C for 15 s, 60 °C for 30 s, and 72 °C for 40 s. Melt curve analysis was performed from 65 to 95 °C in 0.5 °C steps, 10 s for the first step and 5 s for each step thereafter. The CFX Connect Real-Time PCR System (Bio-Rad Laboratories, Hercules, CA, USA) was used to perform real-time qPCR assays. Reactions were run in triplicate and expression levels were analyzed using the relative quantification method modified by [46].

### 4.4. Drugs

In this study, three selective ligands of human GPR18 were used, including two agonists with undisclosed structure (PSB-MZ-1415, EC_50_ 0.019 µM and PSB-MZ-1440, EC_50_ 0.061 µM; chemical details will be shortly published elsewhere) and the antagonist (Z)-2-(3-(6-(4-chlorophenoxy)hexyloxy)benzylidene)-6,7-dihydro-2H-imidazo[2,1-b][1,3]thiazin-3(5H)-one (PSB-CB-27, IC_50_ 0.65 µM). The three ligands were synthetized at the Department of Technology and Biotechnology of Drugs, Faculty of Pharmacy, Jagiellonian University, Kraków, Poland, and identified and characterized in the Department of Pharmaceutical & Medicinal Chemistry, University of Bonn, Germany ([33] and unpublished results).

U46619 ((5Z)-7-{(1R,4S,5S,6R)-6-[(1E,3S)-3-hydroxyoct-1-en-1-yl]-2-oxabicyclo[2.2.1]-heptan-5-yl}hept-5-enoic acid), O-1918 (1,3-dimethoxy-5-methyl-2-[(1R,6R)-3-methyl-6-prop-1-en-2-ylcyclohex-2-en-1-yl]benzene), NAGly, and Abn-CBD were purchased from Tocris Bioscience (Bristol, UK). Stock solutions of these substances were made to 10 mM in ethanol; their final concentrations were prepared by dilutions with deionized water, which led to final concentrations of ethanol of <0.7% *v*/*v*. Stock solutions of PSB-MZ-1415, PSB-MZ-1440, and PSB-CB-27 were made only to 10 mM in dimethyl sulfoxide (DMSO). The final concentration of DMSO was <1.0% *v*/*v*.

### 4.5. Calculations and Statistical Analysis

Results are given as the mean ± SEM of n (number of patients). The relaxation elicited by the GPR18 agonists (NAGly, Abn-CBD, PSB-MZ-1415, PSB-MZ-1440) or the appropriate solvent was expressed as a percentage of the precontraction induced by U46619.

GraphPad Prism 5.0 software (San Diego, CA, USA) was used to plot the mean data as sigmoidal concentration-response curves (CRCs):

$$Y=Bottom+(Top-Bottom)/(1+10^{(}{{}^{LogEC_{50}}}^{-}{{}^{X}}^{)})$$

where *X* is the logarithm of the molar concentration of the agonist and *Y* is the percent response. The curves were used to determine potency (pEC_50_ = −logEC_50_ or pEC_25_ = −logEC_25_, where EC_50_ or EC_25_ is the concentration (M) of the agonist that produced 50 or 25% of the maximal effect (R_max_)) [47].

Antagonist- or endothelium removal-induced rightward shifts of the CRCs relative to the control curve were calculated on the basis of the EC_25_ values. The antagonistic potency (apparent pA_2_) of PSB-CB-27 or O-1918 against GPR18 agonists was calculated from the equation: apparent pA_2_ = log (CR − 1) − log[B], where [B] is the molar concentration of the antagonist and CR is the concentration ratio of the EC_25_ values of GPR18 agonists in the presence and absence of an antagonist.

When two or more treatment groups were compared to the same control, a one-way analysis of variance (ANOVA) followed by Dunnett’s test was used. Post hoc tests were run only if F achieved the necessary level of statistical significance and there was no significant variance inhomogeneity. Student’s *t*-test for unpaired data was used when two groups (one treatment and one control group) were compared. Data were analyzed using GraphPad Prism version 5.00 for Windows, GraphPad Software (La Jolla, CA, USA). Differences were considered to be statistically significant at *p* < 0.05.

## 5. Conclusions

In conclusion, the following facts lend further support to the role of GPR18 in isolated human pulmonary arteries: (i) Two new agonists, PSB-MZ-1415 and PSB-MZ-1440, relax hPAs, (ii) the vasodilatory effect of these agonists is inhibited by the new GPR18 antagonist PSB-CB-27, and (iii) expression of *gpr18* mRNA is shown. GPR18, the activity of which is reduced by endothelial denudation and is lower in hPAs from male, hypercholesterolemic, or smoking patients, may become a new target for the treatment of pulmonary arterial hypertension.

## Figures and Tables

**Table 1:** Vasodilatory effects of NAGly, Abn-CBD, PSB-MZ-1415, and PSB-MZ-1440 on U46619 (0.01 µM)-precontracted isolated human pulmonary arteries (pEC_25_, pEC_50_), interaction with PSB-CB-27 and O-1918, and effect of endothelial removal.

Group	*n*	Contraction (mN)	pEC_25_	pEC_50_	R_max_ (%)
**NAGly**	13	10.8 ± 2.5	5.8 ± 0.1	5.1 ± 0.1	92.5 ± 5.6
+PSB-CB-27 (1 μM)	7	9.9 ± 1.3	5.2 ± 0.1 ***^a^	N.D.	N.D.
+PSB-CB-27 (10 μM)	7	10.5 ± 1.8	4.9 ± 0.1 ***^a^	N.D.	N.D.
+O-1918 (10 μM)	7	9.8 ± 2.1	4.7 ± 0.1 ***^a^	N.D.	N.D.
**Abn-CBD**	5	10.6 ± 2.5	6.2 ± 0.2	5.5 ± 0.1	97.4 ± 4.1
+PSB-CB-27 (10 μM)	7	9.7 ± 1.9	4.8 ± 0.1 ***^b^	N.D.	N.D.
Ethanol	7	8.9 ± 2.6	N.D.	N.D.	4.8 ± 4.1 ***^b^
**PSB-MZ-1415**	10	9.9 ± 1.2	5.8 ± 0.2	5.2 ± 0.2	100.3 ± 5.9
+PSB-CB-27 (10 μM)	6	10.2 ± 1.3	4.6 ± 0.1 ***^a^	N.D.	N.D.
+O-1918 (10 μM)	6	9.8 ± 1.8	4.9 ± 0.2 **^a^	N.D.	N.D.
+Endothelium	3	10.3 ± 1.1	5.9 ± 0.1	5.0 ± 0.1	N.D.
−Endothelium	3	11.3 ± 1.2	4.8 ± 0.1 **^b^	N.D.	N.D.
**PSB-MZ-1440**	8	10.3 ± 2.0	6.2 ± 0.2	4.9 ± 0.1	106.5 ± 5.3
+PSB-CB-27 (10 μM)	6	9.5 ± 1.2	4.8 ± 0.1 ***^b^	N.D.	N.D.
DMSO	10	10.5 ± 1.8	N.D.	N.D.	4.9 ± 4.0 ***^b^

Data are expressed as the mean ± SEM and represent contractile responses to U46619 (0.01 µM). n, number of patients; ** *p* < 0.01, *** *p* < 0.001; compared to the respective control as determined by ^a^ one-way ANOVA followed by Dunnett’s post hoc test or ^b^ Student’s *t*-test. N.D., not determined.

**Table 2:** Apparent pA_2_ values of PSB-CB-27 and O-1918 for their interaction with NAGly, Abn-CBD, PSB-MZ-1415, and PSB-MZ-1440.

	NAGly	Abn-CBD	PSB-MZ-1415	PSB-MZ-1440
PSB-CB-27	6.2	6.3	6.2	6.3
O-1918	6.1	N.D.	5.8	N.D.

Values were calculated from the concentration-response curves shown in Figure 2 and Figure 3 on the basis of the pEC_25_ values obtained in the presence and absence of the antagonists. The concentration of the antagonists was 10 µM. PSB–CB–27 was studied against NAGly also at 1 µM, and the pA_2_ given in the table represents the sum of both single pA_2_ values. N.D., not determined.

**Table 3:** Patient characteristics, diagnosis, types of operations, medications and post hoc analysis of the effect of sex and co-morbidities on PSB-MZ-1415- and NAGly-mediated vasorelaxation in isolated human pulmonary arteries.

Characteristics				Vasodilatory Effect of:					
				PSB-MZ-1415			NAGly		
	N	Range	Mean ± SEM	*n*	pEC_50_	R_max_ (%)	*n*	pEC_50_	R_max_ (%)
Ethnicity (Polish white)	28								
Male	19			6	5.0 ± 0.1	103.3 ± 5.4	9	5.2 ± 0.1	98.4 ± 5.2
Female	9			4	5.5 ± 0.1 **	95.9 ± 10.4	5	5.0 ± 0.1	95.7 ± 10.6
Age (years)	28	51–79	65.2 ± 1.9						
BMI (kg/m^2^)	28	18.2–34.1	25.1 ± 0.8						
Non-smokers	7			4	5.3 ± 0.1	107.3 ± 4.2	3	5.5 ± 0.2	105.0 ± 8.1
Smokers	21			6	5.1 ± 0.1	95.9 ± 9.4	11	4.9 ± 0.1 *	89.9 ± 6.0
0–10 cigarettes per day	5								
10–20 cigarettes per day	16								
Systolic blood pressure (mmHg)	28	109–152	128.5 ± 2.8						
Diastolic blood pressure (mmHg)	28	64–95	73.1 ± 1.8						
No heart diseases	14			5	5.2 ± 0.1	97.9 ± 10.6	6	5.0 ± 0.2	99.2 ± 7.0
Heart diseases	14			5	5.2 ± 0.1	102.8 ± 6.5	8	5.0 ± 0.1	88.6 ± 5.9
Normocholesterolemia	19			3	5.2 ± 0.1	95.3 ± 8.3	9	5.2 ± 0.1	99.4 ± 6.2
Hypercholesterolemia	9			6	5.0 ± 0.1	101.6 ± 9.3	5	4.6 ± 0.1 **	81.9 ± 7.5
Arterial hypertension	9								
Diabetes mellitus	4								
Reason for surgery Operation Tumor staging Medications	Cancer (27 patients), tuberculoma (1) Right upper lobectomy (10), right middle lobectomy (1), right lower lobectomy (7), left upper lobectomy (3), left lower lobectomy (5), left pneumonectomy (1) IA (7), IB (2), IIA (4), IIB (10), IIIA (4) α_1_-Adrenoceptor antagonist (3), ACE inhibitors (8), β blockers (7), statins (9), calcium channel blocker (4), potassium channel blocker (1), diuretics (5), hypoglycemic medication (4), anticoagulants (4), protein pump inhibitors (1)								

Values of pEC_50_ and R_max_ for vasodilatory effects of PSB-MZ-1415 and NAGly are based on the concentration-response curves in Figure 4 and represent mean percentage relaxations of the isometric contraction induced by U46619 and with the standard error of the mean (SEM) fit to non-linear regression (curve fit). N, number of all patients; *n*, the number of patients selected for the respective studies with PSB-MZ-1415 and NAGly. * *p* < 0.05; ** *p* < 0.01; compared to male, non-smokers and normocholesterolemic patients, respectively, as determined by Student’s *t*-test for unpaired data. ACE, angiotensin-converting enzyme; BMI, body mass index; pTNM classification (VIIIth edition).

## Data Availability

The data presented in this study are available on request from the corresponding author. The data are not publicly available due to privacy.

## Abbreviations

Abn-CBD	abnormal cannabidiol
AEA	anandamide
CRC	concentration-response curve
DMSO	dimethyl sulfoxide
hPAs	human pulmonary arteries
NAGly	*N*-arachidonyl-glycine
PSB-MZ-1415	GPR18 agonist
PSB-MZ-1440	GPR18 agonist
PSB-CB-27	GPR18 antagonist
THC	Δ^9^-tetrahydrocannabinol
RVLM	rostral ventrolateral medulla
